# Molecular network-based identification of competing endogenous RNAs in bladder cancer

**DOI:** 10.1371/journal.pone.0220118

**Published:** 2019-08-01

**Authors:** Wei-dong Jiang, Ping-cheng Yuan

**Affiliations:** Department of Urology and Hubei Key Laboratory of Kidney Disease Pathogenesis and Intervention, Huangshi Central Hospital, Affiliated Hospital of Hubei Polytechnic University, Edong Healthcare Group, Huangshi, China; University of Calgary, CANADA

## Abstract

**Background:**

Circular RNAs (circRNAs) have been shown to interact with microRNAs (miRNA) as competitive endogenous RNAs (ceRNAs) to regulate target gene expression and participate in tumorigenesis. However, the role of circRNA-mediated ceRNAs in bladder cancer (BC) remains unknown. Accordingly, the aim of this study was to elucidate the regulatory mechanisms in BC based on construction of the ceRNA network.

**Methods:**

The RNA expression profiles were obtained from public datasets in the Gene Expression Omnibus (GEO) and The Cancer Genome Atlas (TCGA) database, and were used to establish a circRNA-miRNA-mRNA network. The interactions among proteins were analyzed using the STRING database and hubgenes were extracted using the cytoHubba application. Gene Ontology (GO) and Kyoto Encyclopedia of Genes and Genomes (KEGG) pathway analyses of differentially expressed mRNAs in BC and normal tissue samples were performed to determine the functions of the intersecting mRNAs.

**Results:**

A total of 27 circRNAs, 76 miRNAs, and 4744 mRNAs were found to be differentially expressed between BC and normal tissues. The circRNA-miRNA-mRNA ceRNA network was established based on 21 circRNAs, 14 miRNAs, and 150 mRNAs differentially expressed in BC. We also established a protein-protein interaction network and identified 10 hubgenes, which were used to construct circRNA-miRNA-hubgene regulatory modules. The most enriched biological process GO term was strand displacement (P<0.05), and the homologous recombination and Fanconi anemia pathways were significantly enriched (P<0.05) for the differentially expressed genes in BC.

**Conclusions:**

We screened several dysregulated circRNAs and established a circRNA-associated ceRNA network by bioinformatics analysis. The identified ceRNAs are likely critical in the pathogenesis of BC and may serve as future therapeutic biomarkers.

## Introduction

Bladder cancer (BC) is one of the most commonly diagnosed genitourinary malignancies representing a major threat to public health [1]. Although the current clinical treatment of BC has progressed vastly, the 5-year overall survival (OS) remains unsatisfying, especially for patients with metastatic BC [1, 2]. Furthermore, BC has a high recurrence rate (50%), and 15-40% of cases develop into a muscle-invasive form of the disease [3, 4]. Therefore, it is of great importance to clarify the potential molecular mechanisms that may trigger BC, as well as to identify novel targets for disease treatment, which could in turn improve the prognosis and outcome for patients.

Circular RNAs (circRNAs) are novel non-coding RNAs, and are highly conserved across species [5]. Their name originates from the fact that they form closed ring structures without 5′ caps and 3′ tails, conferring them with resistance to exonucleases providing greater stability than linear RNAs [6, 7]. Accumulating evidence indicates that circRNAs could serve as novel prognostic markers in multiple tumor types, such as hepatocellular carcinoma, gastric cancer, and glioblastoma [8–10]. Leonardo Salmena et al. [11] proposed the competitive endogenous RNA (ceRNA) hypothesis in 2011, which posits that a complex post-transcriptional regulatory network of circRNAs can function as a microRNA(miRNA) sponge by complementary base pairing with targeted miRNA using miRNA response elements, thereby inhibiting the activity of miRNAs in regulating the expression of their downstream target genes to contribute to multiple malignancies. Supporting this hypothesis, Wu et al demonstrated that CEP128 acts as a ceRNA to regulate SOX11 expression by sponging miR-145-5p, thereby reducing its inhibitory effect of miR-145-5p on SOX11 in BC [12]. Furthermore, the circRNA hsa_circ_000984 was shown to be significantly upregulated in colorectal cancer (CRC) tissues and cell lines. Knockdown of hsa_circ_000984 inhibited the proliferation, migration, and invasion in vitro and tumor formation in vivo in CRC cell lines. eCDK6 was a downstream mRNA target of miR-106b, its expression was positively regulated by hsa_circ_000984 and negatively regulated by miR-106b. Thus, the authors concluded that hsa_circ_000984 may act as a ceRNA to regulate CDK6 expression in CRC by sponging miR-106b [13].

**Fig 1:**
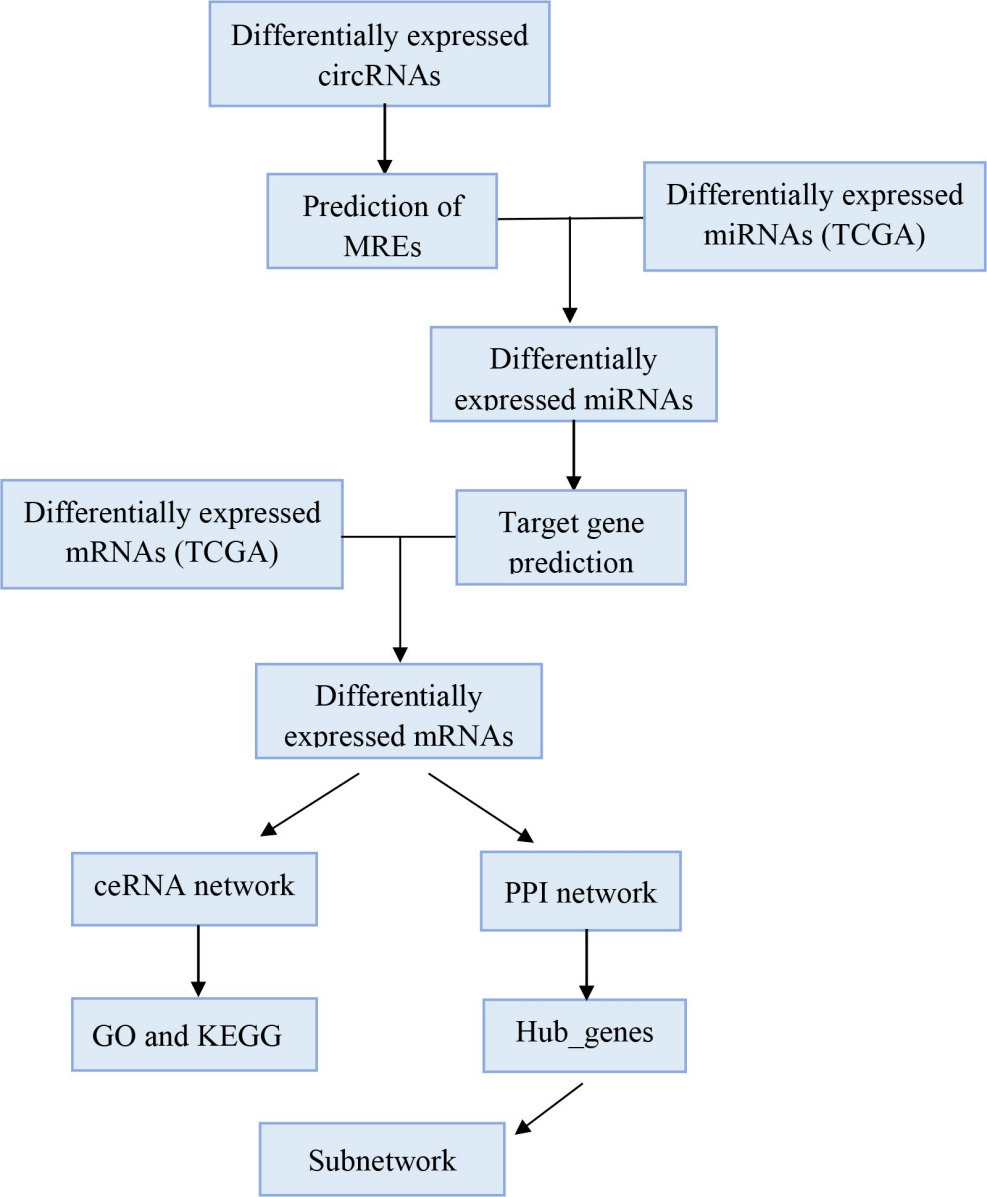
The flowchart of ceRNA network analysis.

In our study, we obtained the circRNA, mRNA, and miRNA expression profiles from the Gene Expression Omnibus (GEO) and The Cancer Genome Atlas (TCGA) database. The flow chart of ceRNA network analysis is shown in Fig 1. After predicting the sponge miRNA of circRNA and miRNA target genes, we successfully constructed a circRNA-miRNA-mRNA network and circRNA-miRNA-hubgene network for BC. To better understand the underlying mechanisms contributing to the pathogenesis, we conducted protein-protein interaction (PPI) and functional enrichment analyses of the differentially expressed genes (DEGs) in the networks. These results can provide further insight into the roles of circRNAs in carcinogenesis, and highlight new treatment targets or biomarkers for BC. Indeed, some of the differentially expressed RNAs identified have been reported in BC previously, but the majority have not, providing new opportunities for novel research directions in this field [14–16]. Given the poor prognosis and high rate of recurrence for patients with BC, these data can offer new opportunities for improving treatment and prevention.

## Materials and methods

### Data acquisition and processing

The circRNA expression profile of the GSE92675 dataset was downloaded from the GEO database, including data from four BC tissues and four normal tissues. The mRNA (414 BC tissues and 19 normal tissues) and miRNA (418 BC tissues and 19 normal tissues) expression profiles were obtained from TCGA database. No ethical approval nor informed consent was required in this study due to the public-available data of GEO. No ethical approval or informed consent was required in this study owing to the use of publicly available data.

### Identification of DEGs

We applied the Limma package to identify differentially expressed circRNAs (DEcircRNAs) with a threshold of |log2 fold change (FC)|> 3 and adjusted P-value < 0.01. The differentially expressed mRNAs (DEmRNAs) and miRNAs (DEmiRNAs) were analyzed using the edgeR package with thresholds of |log2 FC|> 1 and an adjusted P-value < 0.05.

### Construction of the ceRNA network

The circRNA-miRNA interactions were predicted using the Circular RNA Interactome (CircInteractome) (https://circinteractome.nia.nih.gov/) databases. These target miRNAs were further screened according to the DEmiRNAs obtained from TCGA database. In addition, miRNA-targeted mRNAs were retrieved from the miRTarBase and TargetScan databases [17, 18]. Only mRNAs recognized by both databases were considered to be candidate targets, and were intersected with the identified DEmRNAs to screen out the DEmRNAs targeted by the DEmiRNAs. Based on these DEmiRNA-DEcircRNA and DEmiRNA-DEmRNA interactions, we constructed circRNA-miRNA-mRNA regulatory network, which was visualized using Cytoscape 3.7.0 software.

### PPI network construction and analysis

The Search Tool for the Retrieval of Interacting Genes database (Version 10.0, http://string-db.org) was used to predict potential interactions among DEmRNAs. A combined score of > 0.4 was considered significant. Cytoscape 3.7.0 was used for visualization. We used the cytoHubba application to explore the hub genes of the obtained PPI network [19].

### GO annotation and KEGG pathway analysis

The Gene Ontology (GO) annotation and Kyoto Encyclopedia of Genes and Genomes (KEGG) pathway analyses were performed using the clusterProfiler package of R software [20]. P value less than 0.05 were considered to represent statistically significant enrichment of DEGs in pathways or GO terms.

## Results

### Differentially expressed RNAs

**Fig 2:**
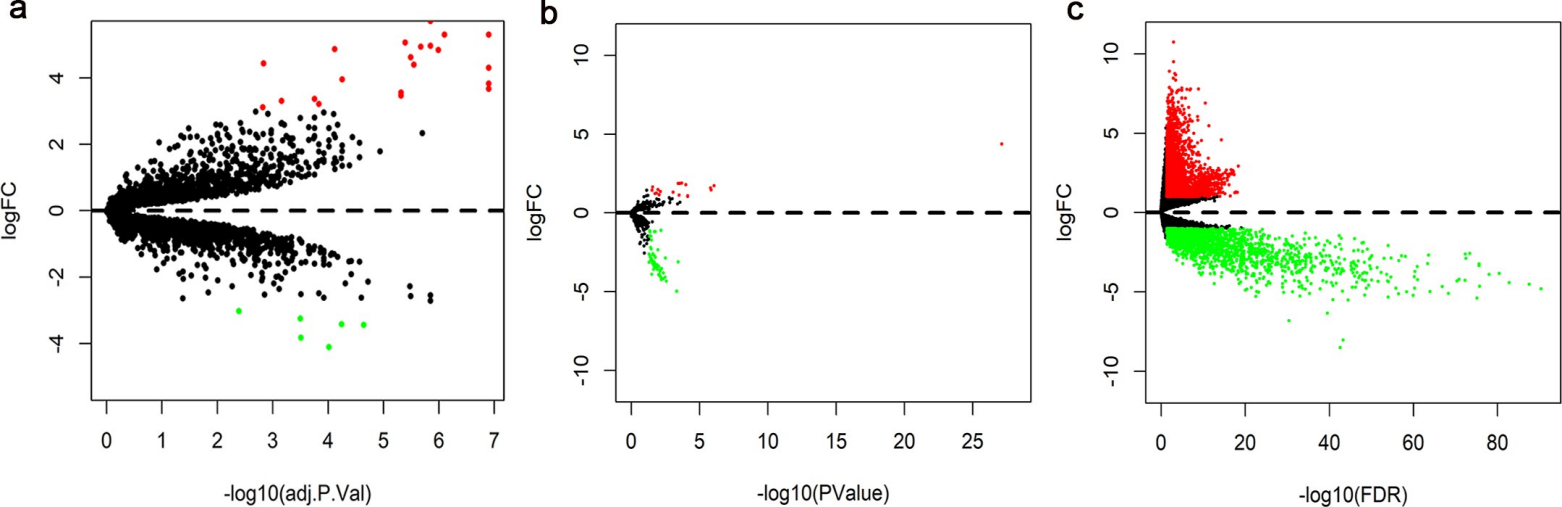
Volcano plot of differentially expressed circRNAs, miRNAs, and mRNAs.

We found 27 DEcircRNAs from the GSE92675 dataset, including 21 up-regulated and 6 down-regulated circRNAs in BC tissues (Fig 2A). We also obtained 76 DEmiRNAs (19 upregulated and 57 downregulated) and 4744 DEmRNAs (2776 upregulated and 1968 downregulated mRNAs) from RNA-Seq data between BC tissues and normal bladder tissues (Fig 2B and 2C).

### Construction of the ceRNA network

**Fig 3:**
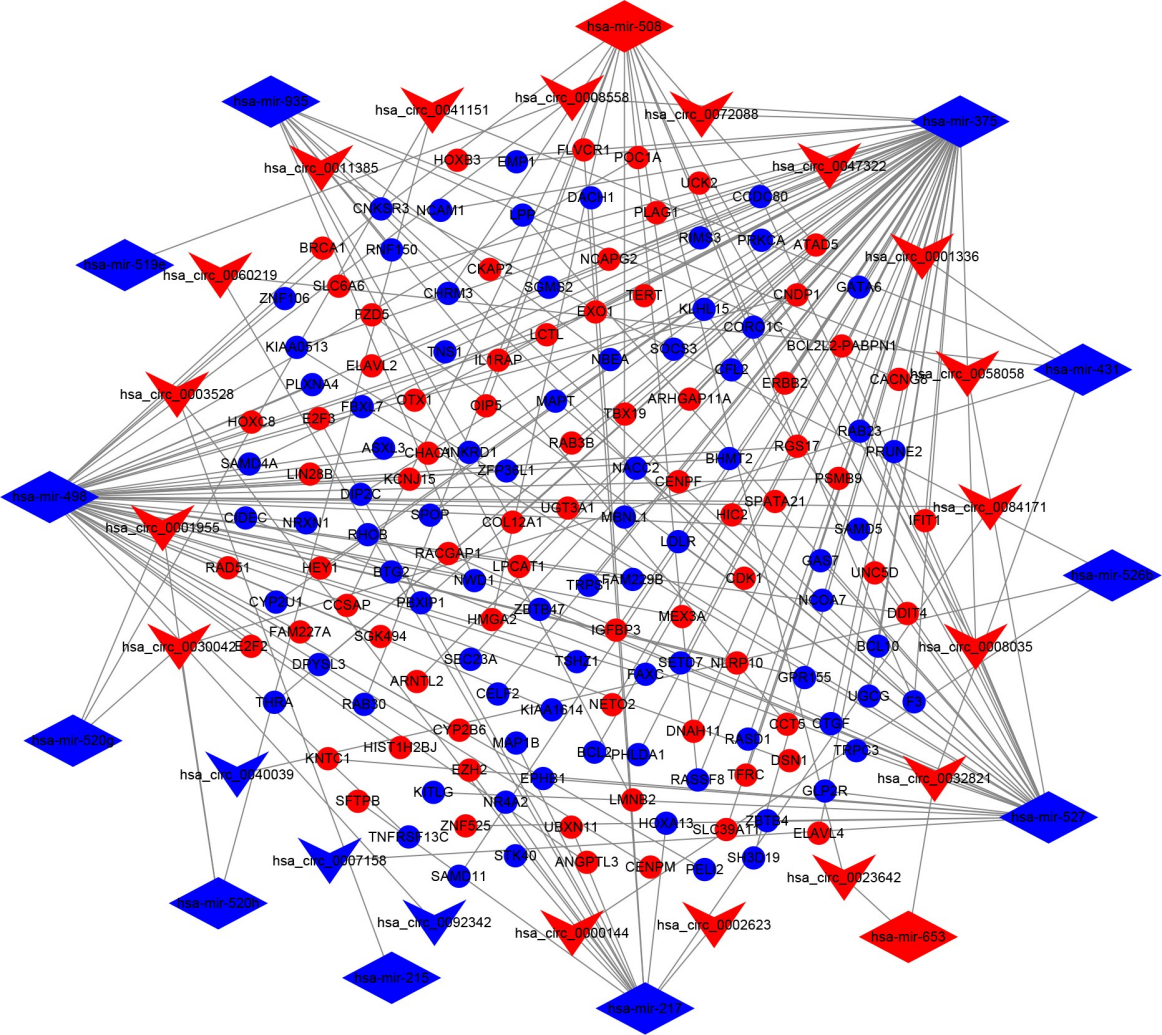
The circRNA-miRNA-mRNA ceRNA network in BC. The network consists of 21 circRNA nodes, 14 miRNA nodes, and 150 mRNA nodes. Vs indicate circRNAs, diamonds indicate miRNA, and ellipses indicate mRNA. The nodes highlighted in red and blue represent up-regulated and down-regulated RNAs, respectively.

To further examine the underlying mechanism of circRNA in mediating mRNA based on miRNA, a ceRNA network was established. We predicted the miRNAs targeted by the 27 DEcircRNAs using the CircInteractome online database. A total of 660 circRNA-miRNA pairs were identified. After intersecting with the DEmiRNAs, 37 circRNA-miRNA pairs, including 21 circRNAs and 14 DEmiRNAs, remained. We further searched for mRNAs targeted by these 14 DEmiRNAs from the miRTarBase and TargetScan databases, and selected those overlapping with the identified DEmRNAs. Ultimately, a total of 150 DEmRNAs were involved in the ceRNA network, along with 21 circRNAs, and 14 miRNAs (Fig 3).

### Construction of the PPI network

**Fig 4:**
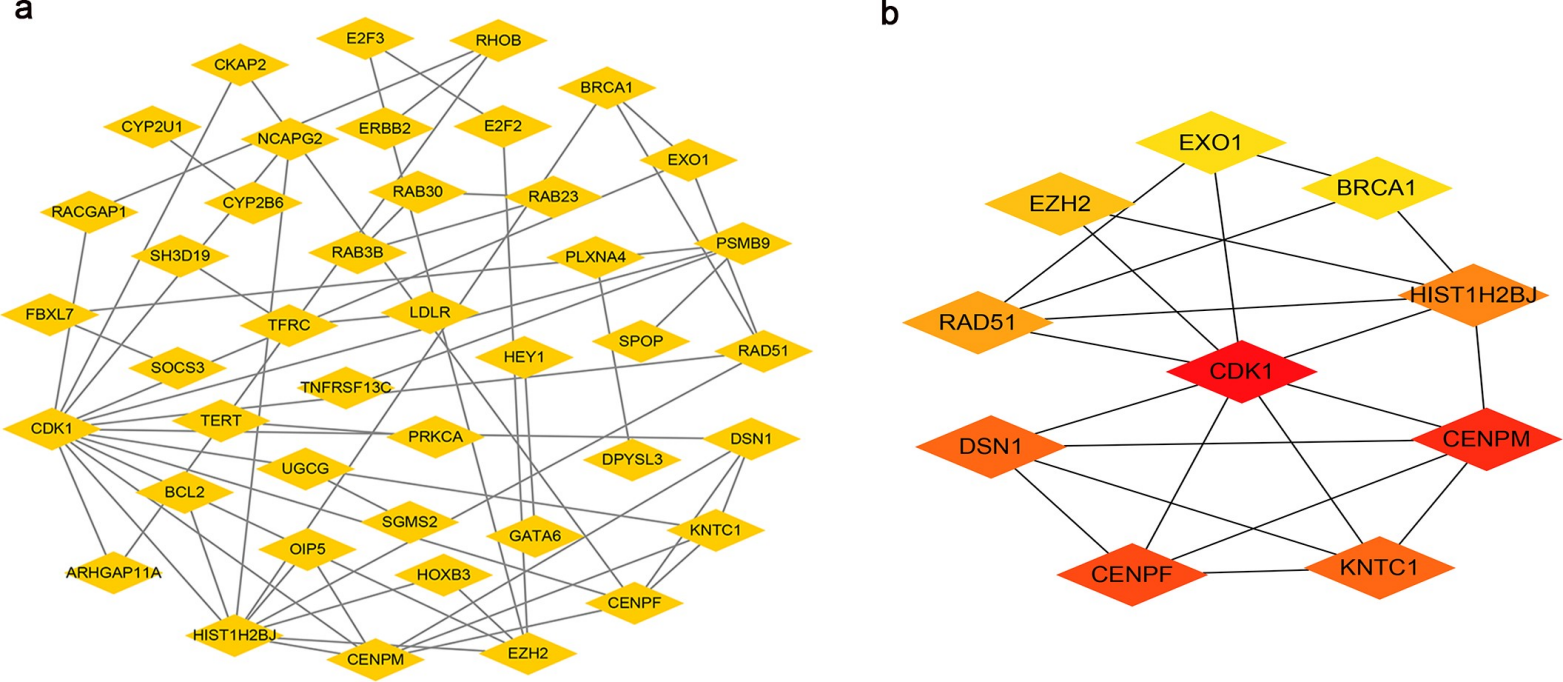
Identification of hubgenes from the PPI network. a. PPI network of 150 genes, consisting of 46 nodes and 53 edges. b. PPI network of 10 hubgenes extracted from a. The node color changes gradually from yellow to red in ascending order according to the log2(foldchange) of genes.

**Fig 5:**
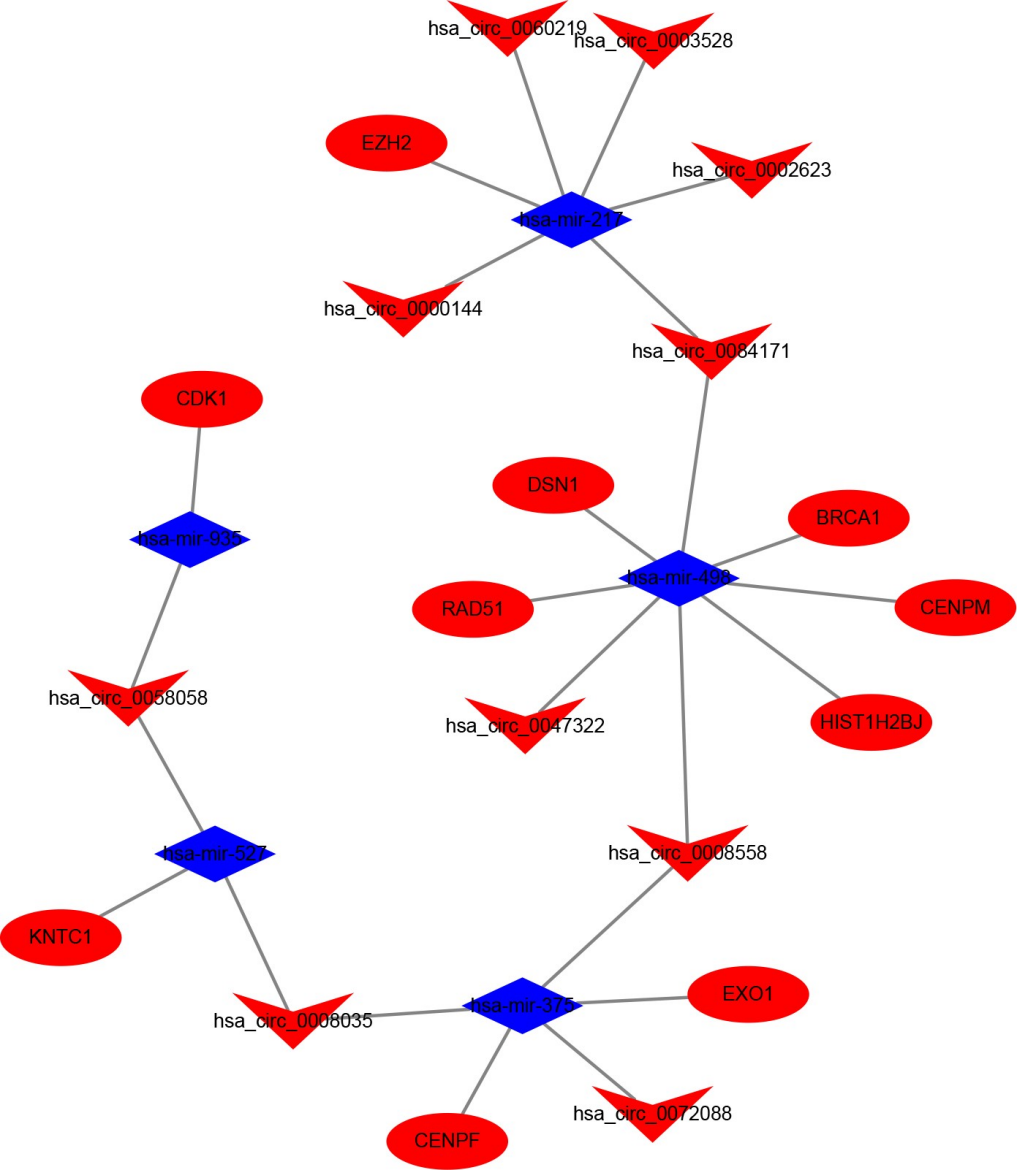
CircRNA-miRNA-hubgene network. The network consists of 10 circRNAs, 5 miRNAs, and 10 hub_genes. Vs indicate circRNAs, diamonds indicate miRNA, and round rectangles indicate mRNA.

After removing unconnected nodes, we established a PPI network that contained 46 nodes and 53 edges (Fig 4A). To explore the hubgenes in the network, indicating a critical role in the process of BC carcinogenesis, the degree and betweenness centrality were evaluated, and the following top 10 hub_genes were extracted using the cytoHubba app: CDK1, CENPM, CENPF, KNTC1, DSN1, HIST1H2BJ, RAD51, EZH2, EXO1, and BRCA1 (Fig 4B). Based on this result, we established a circRNA-miRNA-hub_gene subnetwork, including 31 ceRNA regulatory modules. After excluding modules with inconsistent expression of circRNAs and mRNAs, 29 modules remained (Fig 5).

### Functional assessment

To provide insight into the underlying biological processes and pathways related to the DEmRNAs in the ceRNA network, we performed the GO annotation and KEGG pathway analyses. GO analysis showed that the DEmRNAs were, most significantly enriched in the biological process, cellular components, and molecular function terms strand displacement, condensed chromosome, and endodeoxyribonuclease activity, respectively (P<0.05). The top five GO terms e indicated in Table 1. In addition, the DEmRNAs were significantly enriched in two KEGG pathways: homologous recombination and Fanconi anemia pathway.

**Table 1:** The top 5 GO terms enriched by DEmRNA involved in the ceRNA network.

Categories	Terms	Description	P-value	P-adjusted	Genes	Counts
BP	GO:0000732	strand displacement	3.38E-07	9.57E-05	*RAD51/EXO1/BRCA1*	3
	GO:0045930	negative regulation of mitotic cell cycle	4.37E-07	9.57E-05	*CDK1/CENPF/KNTC1/EZH2/BRCA1*	5
	GO:0007062	sister chromatid cohesion	6.43E-07	9.57E-05	*CENPM/CENPF/KNTC1/DSN1*	4
	GO:0010948	negative regulation of cell cycle process	9.57E-05	3.55E-05	*CDK1/CENPF/RAD51/EZH2/BRCA1*	5
	GO:0007059	chromosome segregation	7.73E-07	9.57E-05	*CENPM/CENPF/KNTC1/DSN1/BRCA1*	5
CC	GO:0000793	condensed chromosome	4.61E-10	2.95E-08	*CENPM/CENPF/KNTC1/DSN1/RAD51/BRCA1*	6
	GO:0098687	chromosomal region	7.72E-09	2.47E-07	*CDK1/CENPM/CENPF/KNTC1/DSN1/RAD51*	6
	GO:0000777	condensed chromosome kinetochore	1.85E-07	3.94E-06	*CENPM/CENPF/KNTC1/DSN1*	4
	GO:0000779	condensed chromosome, centromeric region	2.97E-07	4.75E-06	*CENPM/CENPF/KNTC1/DSN1*	4
	GO:0000776	kinetochore	4.97E-07	6.36E-06	*CENPM/CENPF/KNTC1/DSN1*	4
MF	GO:0004520	endodeoxyribonuclease activity	0.000283	0.018319	*RAD51/EXO1*	2
	GO:0004536	deoxyribonuclease activity	0.000523	0.018319	*RAD51/EXO1*	2
	GO:0004519	endonuclease activity	0.002041	0.045289	*RAD51/EXO1*	2
	GO:0008022	protein C-terminus binding	0.003952	0.045289	*CENPF/RAD51*	2
	GO:0140097	catalytic activity, acting on DNA	0.004286	0.045289	*RAD51/EXO1*	2

## Discussion

In recent years, several computational models have been developed to identify cancer-related non-coding RNAs, and some of them perform well, such as Inductive Matrix Completion for MiRNA–Disease Association prediction (IMCMDA), Matrix Decomposition and Heterogeneous Graph Inference (MDHGI), and Laplacian Regularized Sparse Subspace Learning for MiRNA-Disease Association prediction (LRSSLMDA) [21–24]. However, few computational models predict potential associations between circRNAs and bladder cancer. Our study aims to establish a circRNA-miRNA-mRNA regulatory network.

CircRNAs were first identified in viruses in the 1970s, and then subsequently discovered to be present in human cell lines and the human body [25, 26]. Since circRNAs lack 5′ or 3′ polarities or polyadenylated tails, they are stable and cannot be degraded by RNase-R enzyme [7]. CircRNAs are abundant in eukaryotic cells and show a high degree of conservation, along with structural stability, and a certain degree of organization, timing and disease-specific activity [27, 28]. Based on these features, circRNAs have become research hot_spots, especially with respect to cancer research. Recent, studies have revealed the abundance and function of circRNAs in tumorigenesis [29, 30] and the mechanisms by which circRNAs participate in regulating malignant biological processes [31, 32], demonstrating their potential to serve as biomarkers of malignancies [33–35]. However, the exact role of circRNAs in BC remains largely unclear. This study represents the first attempt at integrating the differentially expressed circRNAs, miRNAs, and mRNAs in BC from public databases to provide a circRNA-miRNA-mRNA regulatory network.

Indeed, accumulating evidence indicates a role of circRNAs in the initiation and progression of BC [36, 37]. Xu et al. [36] analyzed 40 pairs of BC tissue and blood samples and found that circPTK2 was highly expressed in BC. Moreover, they showed that elevated circPTK2 expression could promote the proliferation and migration of BC cells. Chen et al. [37] found that circPRMT5 expression was elevated in BC tissues and was associated with an advanced clinical stage and poor OS of BC patients. They further revealed that circPRMT5 promotes the metastasis of urothelial carcinoma of the bladder through sponging miR-30c to induce the epithelial-mesenchymal transition. In our study, we analyzed BC samples and normal renal samples and they found 21 circRNAs involved in the ceRNA network based on analysis of BC tissues and normal renal samples, including hsa_circ_0000144 and hsa_circ_0023642, whose dysregulated expression has been associated with the pathogenesis and prognosis of BC, indicating their potential as tumor-related biomarker [38, 39]. However, none of the other 19 circRNAs in our ceRNA network have been reported in BC to date.

MiRNAs are non-coding single-stranded RNA molecules that consist of approximately 22 nucleotides [40]. The aberrant expression of miRNAs, which regulate the expression of multiple oncogenes and tumor suppressors, has been widely associated with cancer development [41]. In this study, we identified 14 DEmiRNAs in the ceRNA network. Some researchers have studied the binding of circRNAs to miRNAs and their interactions in BC [14, 42]. Li et al. https://www.ncbi.nlm.nih.gov/pubmed/30382592[14] demonstrated that the circRNA Cdr1as expression inhibited BC cell proliferation, apoptosis, and invasion by sponging miR-135a. Yang et al. [42] indicated that circ-ITCH inhibits BC progression by sponging miR-17/miR-224 and regulating p21 and PTEN expression. Of the 14 miRNAs involved in our ceRNA network, four have been reported to play important roles in the initiation and development of BC, including miR-217, miR-375, and miR-431, and miR-935 [38, 43–45].

To further identify the key circRNAs participating in the regulatory network, we established the PPI network, and screening out 10 hub_genes, followed by construction of the circRNA-miRNA-hubgene network, including 29 ceRNA regulatory modules. To understand the underlying biological processes and pathways between DEmRNAs in the ceRNA network, we performed the functional enrichment analyses. The result indicated that the DEmRNAs were significantly enriched in homologous recombination and Fanconi anemia pathway. Qiao et al. [46] indicated that Imatinib can radiosensitize BC by targeting homologous recombination. In addition, Madubata et al. [47] found that BC patients with Fanconi anemia nonsense variants display a BRCA-deficiency somatic mutation signature. Therefore, the DEmRNAs are involved in many important BC-associated biological functions and metabolic pathways. However, our study presents several limitations. First, the number of samples is not very large. In the future, we will do more analysis based on large samples. Second, the conclusions of our study are only based on the current existing tools and databases. These conclusions e further validated by real experiments. In addition, deep learning, such as graph convolutional neural networks (GCN), convolutional neural networks (CNN), has invaded the field of bioinformatics [48, 49]. In the future, we will try some analysis based on deep learning. Third, the prognostic value of these DEcircRNAs in BC has not been evaluated. In future studies, we will collect more clinical samples validate our findings and further explore the function of these DEcircRNAs using *in vitro* and *in vivo* experiments.

## Conclusions

In this study, we constructed the first ceRNA network in bladder cancer, by identifying DEmiRNAs, DEmRNAs, and DEcircRNAs between BC and normal tissues from data in public databases. Based on these interactions, we identified miRNA and circRNA targets and constructed a protein interaction network to highlight hub genes with likely involvement in BC pathogenesis. We further conducted functional analyses of the DEGs to provide insight into the key biological processes involved in this regulation network. We believe that our study makes a significant contribution to the literature because the roles of circRNAs as potential miRNA "sponges" in several types of cancers are increasingly being emphasized, including BC. Our constructed networks and corresponding modules can serve as a useful guide for further targeted research into the molecular pathogenesis of BC to identify new therapeutic targets and/or biomarkers.

## Supporting information

S1 Table circRNA expression matrix. (TXT)
